# Adult Hippocampal Neurogenesis Can Be Enhanced by Cold Challenge Independently From Beigeing Effects

**DOI:** 10.3389/fnins.2019.00092

**Published:** 2019-03-05

**Authors:** Jong Whi Kim, Kyu Ri Han, Woosuk Kim, Hyo Young Jung, Sung Min Nam, Dae Young Yoo, In Koo Hwang, Je Kyung Seong, Yeo Sung Yoon

**Affiliations:** ^1^Department of Anatomy and Cell Biology, College of Veterinary Medicine, Research Institute for Veterinary Science, Seoul National University, Seoul, South Korea; ^2^Department of Anatomy, College of Veterinary Medicine, Konkuk University, Seoul, South Korea; ^3^Department of Anatomy, College of Medicine, Soonchunhyang University, Asan, South Korea; ^4^Korea Mouse Phenotyping Center, Seoul National University, Seoul, South Korea

**Keywords:** cold challenge, neurogenesis, β adrenergic signaling, hippocampus, CL 316,243

## Abstract

In this study, we investigated the effects of cold challenge on adult hippocampal neurogenesis (AHN) and hippocampal gene expression and whether these are mediated by beigeing of peripheral fat tissues. Cold challenge (6 ± 2°C) for 1 and 4 weeks was found to induce beigeing effects in inguinal white adipose tissue based on hematoxylin and eosin staining as well as uncoupled protein-1 immunohistochemical staining. In the hippocampus, cold challenge for 1 or 4 weeks increased dentate gyrus neurogenesis and expression of genes related to AHN, including notch signaling, G protein-coupled receptor signaling, and adrenergic beta receptor-1. However, this enhancement of neurogenesis and gene expression by cold challenge was not shown by administration of CL 316,243, which induces peripheral beigeing similar to cold challenge but does not cross the blood–brain barrier. These results suggest that cold challenge promotes AHN and central expression of AHN-related, signaling, and β1-adrenergic receptors genes, and that peripheral beigeing by itself is not sufficient to mediate these effects. Considering the increase in AHN and gene expression changes, cold challenge may offer a novel approach to hippocampal modulation.

## Introduction

Adult hippocampal neurogenesis (AHN) is the process of continuous generation of neural stem cells (NSCs) in the subventricular zone of the lateral ventricle and the subgranular zone of the dentate gyrus in the hippocampus (Kempermann et al., 1997; Palmer et al., 1997; Gage, 2000, 2002). A large number of studies have investigated the changes in extrinsic factors during the AHN process, including environmental conditions, diet compositions, and regulatory factors of metabolism (Leuner et al., 2002; Stangl and Thuret, 2009). Metabolic disturbances are also known to cause impairments in AHN. For example, high-fat diet fed mice showed impairments in AHN with an accompanying increase in lipid peroxidation and decrease in brain-derived neurotrophic factor (BDNF) (Park et al., 2010). In addition, a genetically engineered mouse model of type 2 diabetes mellitus (T2DM) and a diet-induced T2DM mouse model showed impaired AHN with dementia (Ramos-Rodriguez et al., 2014). In contrast, metabolic improvements were shown to promote AHN. For example, physical exercise was shown to improve metabolism (Laing et al., 2016), enhanced AHN, synaptic plasticity, and hippocampus-related cognitive function (Brandt et al., 2010; Yau et al., 2011; Vivar et al., 2013). In addition, calorie restriction has been shown to improve AHN while increasing BDNF and other neurotrophins (Lee et al., 2002a,b; Bondolfi et al., 2004).

Several studies have found cold challenge as an extrinsic stimulus that could prevent and improve obesity and DM (Harms and Seale, 2013; Ravussin et al., 2014; Jeremic et al., 2017). As a metabolic improvement factor, cold challenge has been known to improve glucose metabolism and lipid homeostasis in adipose tissue. Metabolic changes induced by cold challenge result from thermogenic effects of adipose tissue (Shabalina et al., 2013). Cold challenge generates heat by activation of brown adipose tissue (BAT) (Nicholls et al., 1978; Gordon, 2012) and beige adipose tissue (BeAT) from white adipose tissue (WAT). This activation is often referred to as ‘beige’ (Ishibashi and Seale, 2010), ‘brite’ (Petrovic et al., 2010), or ‘beigeing’ (Ohno et al., 2012). It has been reported that the “beigeing effects” induced by cold challenge improve metabolic disorders and metabolism in mice (Ravussin et al., 2014). Mild cold challenge (16°C) activated BAT and increased energy expenditure in an experiment on obese and lean people (van Marken Lichtenbelt et al., 2009). In other studies, cycles of cold challenge caused improvement in insulin sensitivity (Lee et al., 2014). The amount of BAT is known to correlate with body mass index, blood glucose levels, age, and sex (Cypess et al., 2009). This regulation is mediated by the activation of uncoupling protein-1 (UCP-1) accompanied by functional upregulation of mitochondria (Shabalina et al., 2013). The epigenetic factors peroxisome proliferator-activated receptor-γ and proliferator-activated receptor γ coactivator 1α are critical regulators of BAT activation and BeAT formation (Lo and Sun, 2013).

Many extrinsic and intrinsic factors are known to induce beige adipocyte recruitment via various mediators (Kajimura et al., 2015). In signaling pathways, BAT activation and beigeing effects are mediated through β3 adrenergic signaling (Peng et al., 2015). CL 316,243, a β3-selective adrenergic agonist, has the potential to activate BAT and form BeAT (Guerra et al., 1998), without any permeability through the brain–blood barrier (Mirbolooki et al., 2011, 2015). Chronic administration of CL 316,243 has been found to increase glucose uptake in brown adipocytes in non-obese rats (de Souza et al., 1997). Formation of BeAT induced by CL 316,243 has also been shown to regulate decrease blood glucose levels while increasing insulin sensitivity (MacPherson et al., 2014). In MKR mice models of T2DM, CL 316,243 administration restored the diabetes-induced downregulation of gene expression related to fatty acid oxidation (Kumar et al., 2015). In transgenic DM mice and rat models, CL 316,243 showed anti-diabetic effects resulting in decreases in glucose, serum fatty acids, and insulin levels (Liu et al., 1998; Ghorbani et al., 2012). To activate BAT and the formation of BeAT by extrinsic stimuli, CL 316,243 has been used for peripheral adipose tissue changes, without direct stimulation to the brain. In this study, we aimed to determine whether AHN is activated or whether beigeing effects are induced by cold challenge.

There are only a few studies about the effect of cold challenge on the brain, specifically the hippocampus. Cold challenge coordinates insulin sensitivity and glucose homeostasis via sympathetic nervous system outflow with thermogenic changes (Nakamura and Morrison, 2007). Correlation and interaction of peripheral adipose tissue with the brain is usually through cytokines from adipose tissue (adipokines; e.g., leptin), for example, the leptin and proopiomelanocortin neurons activated by insulin in the hypothalamus, as well as genetic inhibition of the agouti-related peptide neurons promote beige adipocyte biosynthesis (Ruan et al., 2014; Dodd et al., 2015). In the study of behavioral- and electrophysiological parameter-linked hippocampal function, cold challenge was found to partially improve spatial learning scores but showed impaired long-term potentiation (LTP) (Elmarzouki et al., 2014).

In the present study, we hypothesized that cold challenge can enhance AHN in the hippocampus by the direct effects of cold challenge or by the indirect effects of activation of BAT and BeAT and that this enhanced AHN from cold challenge is independent from the peripheral beigeing effects of BAT and formation of BeAT. We compared the effects of cold challenge and CL 316,243 on hippocampal neurogenesis and gene expression changes related to AHN.

## Materials and Methods

### Experimental Animals

Seven-week-old male C57BL/6N mice were purchased from Japan SLC, Inc. (Shizuoka, Japan). The animals were housed in a specific pathogen-free animal facility at 20 ± 2°C with 60% humidity, a 12 h/12 h light/dark cycle, and *ad libitum* access to food and tap water until the start of the experiment at the age of 8 weeks. The handling and care of the animals conformed to guidelines established in compliance with current international laws and policies (NIH Guide for the Care and Use of Laboratory Animals, NIH Publication No. 85-23, 1985, revised 1996) and was approved by the Institutional Animal Care and Use Committee (IACUC) of Seoul National University (SNU-160111-2). All experiments were conducted with an effort to minimize the number of animals used and the suffering caused by study procedures.

### Cold Challenge Condition

**FIGURE 1:**
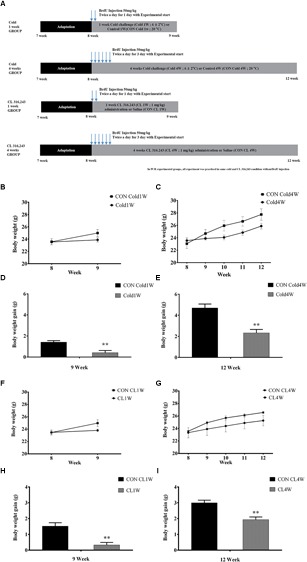
Schematic demonstration of the experimental design **(A)** and effects of cold challenge or CL 316,243 treatment on body weight **(B,C,F,G)** and body weight gain **(D,E,H,I)** during experimental periods. Body weight and body weight gain changed with age and after cold challenge or CL 316,243 treatment for 1 week or 4 weeks (*n* = 5 per group). ^∗∗^ indicates a significant difference compared with the control group (*p* < 0.01). Data are shown as mean ± or + standard error of means (SEM).

At 8 weeks of age, mice were divided into four groups (*n* = 10 in each group): 1 week control group (CON cold1W), 1 week cold challenge group (Cold1W), 4 weeks control group (CON Cold4W), and 4 weeks cold challenge group (Cold4W), depicted in Figure 1A. Cold challenge was conducted by maintaining the cage at 6 ± 2°C, while control mice were kept at 20 ± 2°C in the course of the study (Figure 1A).

### CL 316,243 Administration

At 8 weeks of age, mice were divided into four groups (*n* = 10 in each group); 1 week control group (CON CL1W), 1 week CL 316,243 treated group (CL1W), 4 weeks control group (CON CL4W), and 4 weeks cold challenge group (CL4W) at 20 ± 2°C, depicted in Figure 1A. To induce beigeing in adipose tissues, the animals received subcutaneous injections of 1 mg/kg CL 316,243 every day during the experimental period. The same volume of saline was subcutaneously injected into the mice in the control group for the same duration (Figure 1A).

### Labeling of Newly Generated Cells

To label newly generated cells in the hippocampus, intraperitoneal injection of 5-Bromo-2′-deoxyuridine (BrdU; 50 mg/kg; Sigma-Aldrich, St. Louis, MO, United States) was administered to mice (*n* = 5 in each group) and proceeded as outlined in the scheme in Figure 1A, except for the animals from which samples for polymerase chain reaction (PCR) were to be obtained.

### Tissue Preparation

Animals (*n* = 5 in each group) were anesthetized by intraperitoneal injection of 1 g/kg urethane (Sigma-Aldrich), and perfused transcardially with 0.1 M phosphate-buffered saline (PBS; pH 7.4), followed by 4% paraformaldehyde in 0.1 M PBS. The brains were dissected out and post-fixed in the same fixative for 12 h. The brain tissues were cryoprotected overnight using infiltration with 30% sucrose. Thirty-micrometer thick brain sections were serially sectioned in the coronal plane using a cryostat (Leica, Wetzlar, Germany) and collected in six-well plates containing PBS at -20°C for further processing.

Mouse BAT, iWAT, and epididymal WAT (eWAT) were also dissected and fixed for 48 h in 4% paraformaldehyde. The fixed adipose tissues were dehydrated with graded concentrations of ethyl alcohol for embedding in paraffin. Paraffin-embedded tissues were sectioned on a microtome (Leica, Wetzlar, Germany) into 3 μm coronal sections and mounted onto silane-coated slides.

### Hematoxylin and Eosin Staining

Paraffin sections in each animal (*n* = 10 in each group) were dewaxed with xylene, hydrated with alcohol, immersed in Harris hematoxylin (Sigma-Aldrich) for 5 min, and washed in running tap water for 3 min. Sections were immersed in eosin (Sigma-Aldrich) for 30 s with agitation. Excess dye was washed and sections were differentiated in running tap water for 30 s. Thereafter, the sections were dehydrated serially with alcohol, cleared with xylene, and mounted in Canada balsam (Kanto Chemical, Tokyo, Japan).

### Immunohistochemistry

To obtain accurate data for immunohistochemistry in hippocampal tissue, the free-floating sections from all animals were processed carefully under the same conditions. For each animal, tissue sections were selected between 1.46 and 2.46 mm posterior to the bregma using the mouse atlas by Franklin and Paxinos for reference (Franklin and Paxinos, 2013). Five sections, 180 μm apart, were sequentially treated with 0.3% hydrogen peroxide in PBS for 30 min and 10% normal goat or rabbit serum in 0.05 M PBS for 30 min. They were then incubated with a rabbit anti-Ki-67 antibody (1:1,000; Abcam, Cambridge, United Kingdom) or goat anti-doublecortin (DCX) antibody (1:50; Santa Cruz Biotechnology, Santa Cruz, CA, United States) overnight at 25°C and, subsequently, treated with either a biotinylated goat anti-rabbit IgG or a rabbit anti-goat IgG, and a streptavidin-peroxidase complex (1:200; Vector Labs, Burlingame, CA, United States). Sections were stained following reaction with 3,3′-diaminobenzidine tetrachloride (Sigma-Aldrich) in 0.1 M Tris-HCl buffer (pH 7.2) and then dehydrated and mounted in Canada balsam (Kanto Chemical) onto silane-coated slides.

### Immunofluorescence

For UCP-1 immunofluorescence in the fat tissues, the sections were hydrated and treated with citrate buffer for 30 min at 37°C. They were then incubated with a rabbit anti-UCP-1 (1:1000; Millipore) for 2 h at 25°C, followed by overnight incubation at 4°C. After washing with PBS, the sections were subsequently incubated with Cy3-conjugated goat anti-rabbit IgG (1:100; Jackson ImmunoResearch, West Grove, PA, United States) for 2 h. Thereafter, the slides were mounted with a DAPI-containing mounting medium (Vector Labs) for nuclei staining.

For TBR-2 immunofluorescence, sections were incubated with a rabbit anti-TBR-2 antibody (1:200; Millipore, Temecula, CA, United States) for 2 h at 25°C, followed by overnight incubation at 4°C. After washing with PBS, the sections were subsequently incubated with Cy3-conjugated goat anti-rabbit IgG (1:100; Jackson ImmunoResearch) for 2 h. After, the slides were mounted with DAPI-containing mounting medium (Vector Labs) for nuclei staining.

For BrdU and neuronal nuclei (NeuN) double immuno fluorescence, the sections were treated with 2 N HCl for 30 min at 37°C and were incubated with a mixture of mouse anti-NeuN (1:1000; Millipore) and rat anti-BrdU (1:200; Abd Serotec, Bio-Rad Laboratories, Inc., Grand Island, NY, United States), for 2 h at 25°C, followed by overnight incubation at 4°C. For BrdU and doublecortin (DCX) double immunofluorescence, the sections were treated with 2 N HCl for 30 min at 37°C and were incubated with a mixture of mouse anti-NeuN (1:1000; Millipore) and goat anti-DCX (1:25; Santa Cruz Biotechnology) for 2 h at 25°C, followed by overnight incubation at 4°C. After washing with PBS, the sections were subsequently incubated in a mixture of FITC-conjugated goat anti-mouse IgG (1:100; Jackson ImmunoResearch) and Cy3-conjugated goat anti-rat IgG (1:100; Jackson ImmunoResearch) for BrdU and NeuN double immunofluorescence, or FITC-conjugated rabbit anti-goat IgG (1:100; Vector Labs) and Cy3-conjugated rabbit anti-rat IgG (1:100; Vector Labs) for BrdU and DCX double immunofluorescence for 2 h. Thereafter, the sections were mounted in a DAPI-containing mounting medium (Vector Labs, Burlingame, CA, United States) onto gelatin-coated slides for nuclei staining.

### Microscopic Analysis

Two independent, blinded investigators counted the number of TBR-2, Ki-67, DCX, BrdU/DCX, or BrdU/NeuN-labeled cells in the dentate gyrus at 400× magnification under a light microscope with a Panoramic Scan II (3DHISTECH, Budapest, Hungary). All TBR-2, Ki-67, DCX, BrdU/DCX, or BrdU/NeuN-labeled cells were counted bilaterally in five sections (180 μm apart from each other) across the entire dentate gyrus between 1.46 and 2.46 mm posterior to the bregma, using the mouse atlas by Franklin and Paxinos for reference (Franklin and Paxinos, 2013). For analyzing beigeing effects from cold challenge and CL 316,243 treatment, region of interest (ROI) was measured from UCP-1 immunofluorescence in the ten fat tissues sections using the triangle thresholding methods ImageJ v. 1.50 software (National Institutes of Health) in 512 pixels × 512 pixels at 400×. ROI data were expressed as a percent of total area.

### Quantitative Real-Time PCR

Animals (*n* = 5 in each group) were euthanized by an intraperitoneal injection of 2 g/kg urethane (Sigma-Aldrich), and the brain tissues were cut into 500-μm-thick sections on a vibratome (Leica Microsystems GmbH). Thereafter, the dorsal hippocampus was dissected using a surgical blade and was quickly dissected from the brain. Total RNA from the dorsal hippocampus was extracted using an RNA purification system (Invitrogen), following the manufacturer’s protocol. mRNA was reverse-transcribed using AccuPower CycleScript RT PreMix^®^(Bioneer, Daejeon, South Korea), and a quantitative PCR (qPCR) was performed using SYBR Green in an ABI StepOne Real-Time PCR^®^instrument (Applied Biosystems, Cheshire, United Kingdom). Target gene expression was normalized to that of the control gene (36B4) and relative expression was quantified by the comparative Ct method (ΔΔCt). The primers used for PCR are listed in Table 1. To identify changes in gene expression responding to cold challenge and CL 316,243 administration, the fold change method (1.3-fold as a cutoff value) was used. Genes with a fold change ≥1.3 were thought to be significantly disturbed in response to cold challenge and CL 316,243 administration and were thus selected for further analysis.

**Table 1:** Primers used for qRT-PCR.

		SEQUENCE (5′ → 3′)		SEQUENCE (5′ → 3′)
Ache	F	CTCCCTGGTATCCCCTGCATA	R	GGATGCCCAGAAAAGCTGAGA
Ascl-1	F	GCAACCGGGTCAAGTTGGT	R	GTCGTTGGAGTAGTTGGGGG
Adrb1	F	CTCATCGTGGTGGGTAACGTG	R	ACACACAGCACATCTACCGAA
Adrb2	F	GGGAACGACAGCGACTTCTT	R	GCCAGGACGATAACCGACAT
Adrb3	F	GGCCCTCTCTAGTTCCCAG	R	TAGCCATCAAACCTGTTGAGC
Bdnf	F	TCATACTTCGGTTGCATGAAGG	R	AGACCTCTCGAACCTGCCC
Bmp2	F	GGGACCCGCTGTCTTCTAGT	R	TCAACTCAAATTCGCTGAGGAC
Bmp4	F	TTCCTGGTAACCGAATGCTGA	R	CCTGAATCTCGGCGACTTTTT
Bmp8	F	ACATGCAGCGTGAAATCCTG	R	GCGTGGTATAGGTCCAACATGA
Creb1	F	AGCAGCTCATGCAACATCATC	R	AGTCCTTACAGGAAGACTGAACT
Chrm2	F	TGGTTTGGCTATTACCAGTCCT	R	CTGAAGGTGGCGGTTGACTT
Drd2	F	ACCTGTCCTGGTACGATGATG	R	GCATGGCATAGTAGTTGTAGTGG
Erbb2	F	GAGACAGAGCTAAGGAAGCTGA	R	ACGGGGATTTTCACGTTCTCC
Fgf2	F	GCGACCCACACGTCAAACTA	R	TCCCTTGATAGACACAACTCCTC
Grin1	F	AGAGCCCGACCCTAAAAAGAA	R	CCCTCCTCCCTCTCAATAGC
Hdac4	F	CTGCAAGTGGCCCCTACAG	R	CTGCTCATGTTGACGCTGGA
Hes1	F	CCAGCCAGTGTCAACACGA	R	AATGCCGGGAGCTATCTTTCT
Heyl	F	CAGCCCTTCGCAGATGCAA	R	CCAATCGTCGCAATTCAGAAAG
Mdk	F	GAAGAAGGCGCGGTACAATG	R	GAGGTGCAGGGCTTAGTCA
Neurod1	F	ATGACCAAATCATACAGCGAGAG	R	TCTGCCTCGTGTTCCTCGT
Neurog1	F	CCAGCGACACTGAGTCCTG	R	CGGGCCATAGGTGAAGTCTT
Neurog2	F	AACTCCACGTCCCCATACAG	R	GAGGCGCATAACGATGCTTCT
Notch1	F	GATGGCCTCAATGGGTACAAG	R	TCGTTGTTGTTGATGTCACAGT
Nrcam	F	GGAAGTCGAACACCTTCAGAC	R	AGTTCATTGCGTTGACAGGAG
Nrg1	F	ATGGAGATTTATCCCCCAGACA	R	GTTGAGGCACCCTCTGAGAC
Ntf3	F	GGAGTTTGCCGGAAGACTCTC	R	GGGTGCTCTGGTAATTTTCCTTA
Olig2	F	TCCCCAGAACCCGATGATCTT	R	CGTGGACGAGGACACAGTC
Pax3	F	CCGGGGCAGAATTACCCAC	R	GCCGTTGATAAATACTCCTCCG
Pax5	F	CCATCAGGACAGGACATGGAG	R	GGCAAGTTCCACTATCCTTTGG
Pax6	F	TACCAGTGTCTACCAGCCAAT	R	TGCACGAGTATGAGGAGGTCT
Pou4f1	F	CGCGCAGCGTGAGAAAATG	R	CGGGGTTGTACGGCAAAATAG
S100b	F	TGGTTGCCCTCATTGATGTCT	R	CCCATCCCCATCTTCGTCC
Sod1	F	AACCAGTTGTGTTGTCAGGAC	R	CCACCATGTTTCTTAGAGTGAGG
Sox2	F	GCGGAGTGGAAACTTTTGTCC	R	CGGGAAGCGTGTACTTATCCTT
Tgfb1	F	CTCCCGTGGCTTCTAGTGC	R	GCCTTAGTTTGGACAGGATCTG
36B4	F	CGACCTGGAAGTCCAACTAC	R	ATCTGCTGCATCTGCTTG

### Statistical Analysis

Statistical analyses were performed using the SPSS version 20.1 (IBM Corporation, Armonk, NY, United States). Body weight and body weight gain data were compared using independent *T*-test analysis. IHC and IF data were compared using one-way or two-way analysis of variance (ANOVA) followed by a Tukey’s honest significant difference *post hoc* analysis. qRT-PCR data were compared using independent *T*-test analysis.

## Results

### The Effects of Cold Challenge and CL 316,243 Administration on Body Weight and Body Weight Gain

At 8 weeks of age, the animals in each group had similar body weights (Figure 1B,C,F,G). Body weight increased with age in all groups; however, body weight showed no significant changes in the Cold1W, Cold4W, CL1W, and CL4W groups when compared with that in their respective control groups (Figure 1B,C,F,G). In contrast, the body weight gains seen in the Cold1W, Cold4W, CL1W, and CL4W groups were significantly less than those in their respective control groups (Figure 1D,E,H,I). Statistical data of body weight gains were *t*(8) = 3.881 and *p* < 0.01 in Cold1W (Figure 1D), *t*(8) = 4.748 and *p* < 0.01 in CL1W (Figure 1E), *t*(8) = 4.392 and *p* < 0.01 in Cold4W (Figure 1H), and *t*(8) = 4.466 and *p* < 0.01 in CL4W (Figure 1I).

### Effects of Cold Challenge and CL 316,243 Treatment on Morphological Changes in BAT, iWAT, and eWAT Caused by Beigeing

**FIGURE 2:**
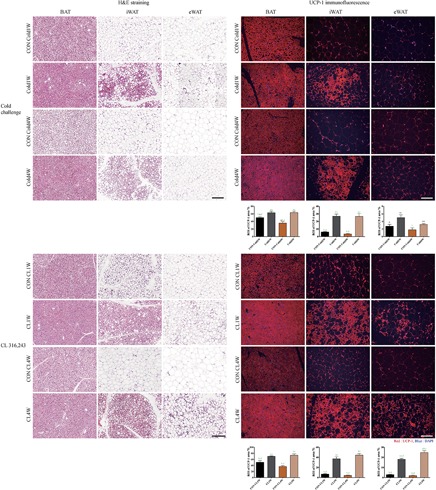
Effects of cold challenge or CL 316,243 treatment for 1 week or 4 weeks on the morphology and UCP-1 immunofluorescence of BAT, iWAT, and eWAT. Representative images of hematoxylin and eosin staining of BAT, iWAT, and eWAT, after 1 week or 4 weeks of cold challenge or CL 316,243 treatment. Hematoxylin and eosin staining show that lipid accumulation is decreased in BAT of the Cold1W, Cold4W, CL1W, and CL4W groups when compared with that in their control groups. In addition, UCP-1 immunoreactive structures are prominently found in BAT and iWAT of the Cold1W, Cold4W, CL1W, and CL4W groups when compared with those in the CON Cold1W, CON Cold4W, CON CL1W, and CON CL4W groups, respectively. Scale bar = 100 μm. Region of interest (ROI) is the region of interest of UCP-1 immunofluorescent structures in fat tissues is also shown. ‘a’ indicates a significant difference when compared with the CON Cold1W group (*p* < 0.05); ‘b’ indicates a significant difference when compared with the Cold1W group (*p* < 0.05); ‘c’ indicates a significant difference when compared with the CON Cold4W group (*p* < 0.05); ‘d’ indicates a significant difference when compared with the Cold4W (*p* < 0.05) group; and green ‘a’,’b’,’c’, and ‘d’ indicates a significant difference (*p* < 0.01). Data are shown as mean + SEM.

For histological analysis of morphological changes of BAT, iWAT, and eWAT, hematoxylin and eosin staining was performed. In the Cold1W and Cold4W groups, less lipid accumulation was found in the BAT when compared with their respective control groups. In addition, iWAT in the Cold1W and Cold4W groups showed beigeing morphology with decreased lipid accumulation when compared with their respective control groups. However, eWAT did not show remarkable morphological changes, although less lipid accumulation was found in eWAT of the Cold1W and Cold4W groups. Similarly, less lipid accumulation was found in the BAT of the CL 1W and CL 4W groups when compared with their respective control groups. In addition, in the CL 1W and CL 4W groups, iWAT and eWAT showed less lipid accumulation, as well as beigeing-induced morphological changes when compared to their respective control groups (Figure 2).

For confirmation of beigeing effects after cold challenge and CL 316,243 treatment in BAT, iWAT, and eWAT, UCP-1 immunofluorescent staining was conducted after cold challenge or CL 316,243 treatment for 1 and 4 weeks and ROI was measured to quantitatively analyze effects of cold challenge and CL 316,243 on adipocytes. In the Cold1W and Cold4W groups, UCP-1 immunoreactivity was observed in BAT and ROI was significantly increased when compared with the BAT of CON Cold1W and CON Cold4W groups. In the ROI of UCP-1 in BAT from Cold1W and Cold4W groups, statistical data of between subject effects were *F*(1.20) = 100.531 and *p* < 0.01 from cold, *F*(1.20) = 9.578 and *p* < 0.01 from duration, and *F*(1.20) = 10.606 and *p* < 0.01 from cold and duration. The iWAT UCP-1 immunoreactivity in the CON Cold1W and CON Cold4W groups was diffusely found in the cytoplasm of adipocytes and this distribution pattern was similarly observed in the eWAT of the CON Cold1W, CON Cold4W, Cold1W, and Cold4W groups. However, in the iWAT of the Cold1W and Cold4W groups, UCP-1 immunoreactivity was aggregated and prominent and showed similar morphology to BeAT with increased ROI of UCP-1 (Figure 2). In the ROI of UCP-1 in iWAT from Cold1W and Cold4W groups, statistical data of between subject effects were *F*(1.20) = 86.321 and *p* < 0.01 from cold, *F*(1.20) = 0.368 and *p* = NS from duration, and *F*(1.20) = 0.245 and *p* = not significant (NS) from cold and duration. In the ROI of UCP-1 in eWAT from Cold1W and Cold4W groups, statistical data of between subject effects were *F*(1.20) = 12.013 and *p* < 0.01 from cold, *F*(1.20) = 6.688 and *p* < 0.05 from duration, and *F*(1.20) = 0.732 and *p* = NS from cold and duration.

In the CL1W and CL4W groups, UCP-1 immunoreactivity was strongly detected in the BAT, compared with BAT in the control groups, with increased ROI (Figure 2). In the ROI of UCP-1 in BAT from CL1W and CLW groups, statistical data of between subject effects were *F*(1.20) = 56.635 and *p* < 0.01 from CL 316,243, *F*(1.20) = 1.889 and *p* = NS from duration, and *F*(1.20) = 5.287 and *p* < 0.05 from CL 316,243 and duration. UCP-1 immunoreactivity in iWAT and eWAT of the CON CL1W and CON CL4W groups were detected in the smaller and peripheral located cytoplasm of adipocytes. However, in the CL1W and CL4W groups, UCP-1 immunoreactive structures were abundantly observed in iWAT and eWAT when compared with those in the control groups with increased ROI (Figure 2). In the ROI of UCP-1 in iWAT from CL1W and CL4W groups, statistical data of between subject effects were *F*(1.20) = 182.241 and *p* < 0.01 from CL 316,243, *F*(1.20) = 0.809 and *p* = NS from duration, and *F*(1.20) = 3.414 and *p* = NS from CL 316,243 and duration. In the ROI of UCP-1 in eWAT from CL1W and CL4W groups, statistical data of between subject effects were *F*(1.20) = 313.591 and *p* < 0.01 from CL 316,243, *F*(1.20) = 7.067 and *p* < 0.05 from duration, and *F*(1.20) = 12.666 and *p* < 0.01 from CL 316,243 and duration. UCP-1 immunoreactivity was most abundant in the iWAT of the CL1W and CL4W groups when compared with other control groups or with eWAT (Figure 2).

### Cold Challenge, Not CL 316,243 Treatment, for 1 Week or 4 Weeks Increased NPCs in the Hippocampus

**FIGURE 3:**
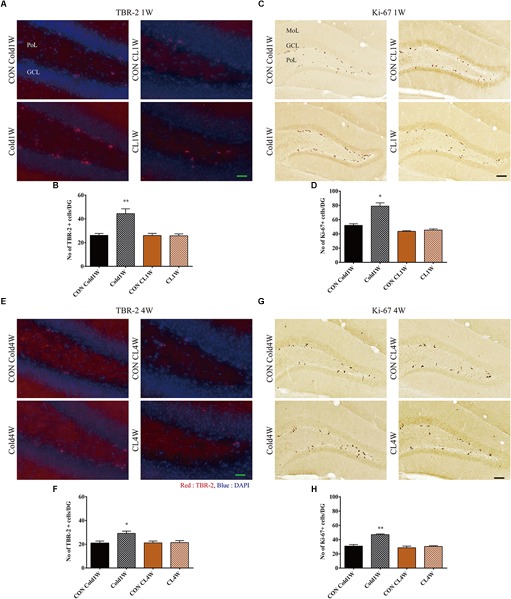
Effects of cold challenge or CL 316,243 treatment for 1 week or 4 weeks on the TBR-2 and Ki-67 positive cells in the dentate gyrus. Note that the TBR-2 and Ki-67 positive cells are abundant in the Cold1W and Cold4W groups when compared with that in the CON Cold1W and CON Cold 4W groups, respectively **(A,C,E,G)**. However, the CON CL1W, CL1W, CON CL4W, and CL4W groups show similar populations of TBR-2 and Ki-67 positive cells in the dentate gyrus **(A,C,E,G)**. GCL, granule cell layer; MoL, molecular layer; PoL, polymorphic layer. Quantitative analysis of TBR-2 and Ki-67 positive cells per section in the CON Cold1W, Cold1W, CON Cold4W, Cold4W, CON CL1W, CL1W, CON CL4W, and CL4W groups **(B,D,F,H)** (*n* = 5 per group); ^∗^ indicates a significant difference when compared with the CON group (*p* < 0.05); and ^∗∗^ indicates a significant difference when compared with the CON group (*p* < 0.01). Data are shown as mean + SEM. Black scale bar = 100 μm and green scale bar = 25 μm.

To analyze the correlation between beigeing effects and NPCs, population changes of NPCs were observed in the hippocampus with TBR-2. In all groups, TBR-2 immunoreactivity was mainly detected in the sub-granular zone of the dentate gyrus (Figure 3A,E). There were significantly higher populations of TBR-2 positive cells in the Cold1W group when compared with the CON Cold1W group (Figure 3A). The mean number of TBR-2 positive cells was significantly increased in the Cold1W group (44.40 ± 4.11) when compared with the CON Cold1W group (26.20 ± 1.53) (Figure 3B). In contrast, TBR-2 immunoreactivity in the CL1W group did not show any difference in NPCs numbers when compared with their respective control group (Figure 3A). The mean number of TBR-2 positive cells was 26.00 ± 1.79 in the CON CL1W and 25.80 ± 1.59 in the CL1W mice. In the number of TBR-2 positive cells after cold challenge or CL 316,243 treatment for 1 week, statistical data of between groups were *F*(3.20) = 13.579 and *p* < 0.01. Additionally, there were significantly higher populations of TBR-2 positive cells in the Cold4W group when compared with the CON Cold4W group (Figure 3E). The mean number of TBR-2 positive cells was 21.00 ± 1.67 in the CON Cold4W mice and 29.20 ± 1.88 in the Cold4W mice (Figure 3F). In contrast, TBR-2 immunoreactivity in the CL4W group did not show any difference in NPCs numbers when compared with their respective control group (Figure 3E). In addition, the mean number of TBR-2 positive cells were 21.20 ± 1.52 and 21.20 ± 1.69 in the CON CL4W and CL4W mice, respectively (Figure 3F). In the number of TBR-2 positive cells after cold challenge or CL 316,243 treatment for 4 weeks, statistical data of between groups were *F*(3.20) = 5.555 and *p* < 0.01.

### Cold Challenge, Not CL 316,243 Treatment, for 1 Week or 4 Weeks Increased Proliferating Cells in the Hippocampus

We observed proliferating cells with Ki-67 immunohistochemical staining of the subgranular zone of the dentate gyrus. In all groups, Ki-67 immunoreactivity was found in the subgranular zone of the dentate gyrus (Figure 3C,G). In the Cold1W group, the population of Ki-67-positive cells was significantly increased in the dentate gyrus when compared with the CON Cold1W group (Figure 3C). The mean number of Ki-67 positive cells was 52.00 ± 2.30 in the CON Cold1W mice and 79.00 ± 4.52 in Cold1W mice (Figure 3D). However, there were no differences in the populations of Ki-67 positive cells between the CON CL1W and CL1W groups and the mean number of Ki-67 positive cells was 43.60 ± 0.93 in the CON CL1W mice and 45.40 ± 1.57 in the CL1W mice (Figure 3D). In the number of Ki-67 positive cells after cold challenge or CL 316,243 treatment for 1 week, statistical data of between groups were *F*(3.20) = 36.956 and *p* < 0.01. In addition, cold challenge increased the population of Ki-67 positive cells in the Cold4W group when compared with the CON Cold4W group (Figure 3G). The mean number of Ki-67 positive cells was 31.00 ± 2.25 and 47.00 ± 1.05 in the CON Cold4W and Cold4W mice, respectively (Figure 3H). In addition, the number of Ki-67 positive cells were similarly observed in the CON CL4W and CL4W groups. The mean number of Ki-67 positive cells was 28.60 ± 2.25 in the CON CL4W mice and 30.40 ± 1.03 in the CL4W mice (Figure 3H). In the number of Ki-67 positive cells after cold challenge or CL 316,243 treatment for 4 weeks, statistical data of between groups were *F*(3.20) = 25.898 and *p* < 0.01.

### Cold Challenge, Not CL 316,243 Treatment, for 1 Week or 4 Weeks Increased Neuroblasts in the Hippocampus

**FIGURE 4:**
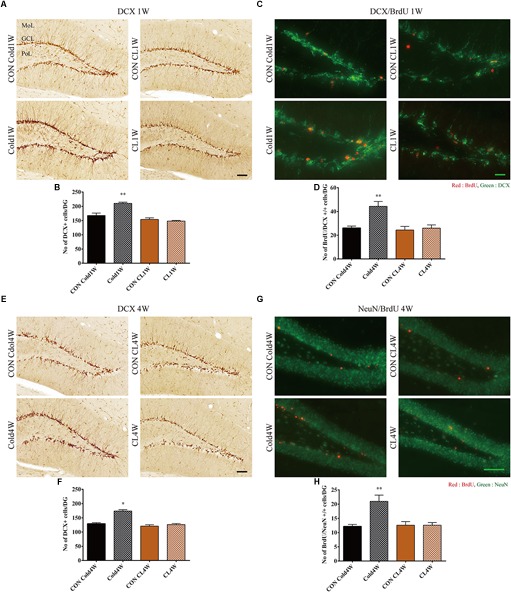
Effects of cold challenge or CL 316,243 treatment for 1 week or 4 weeks on DCX immunoreactive neuroblasts, 1 week on differentiation of proliferating cells into DCX immunoreactive neuroblasts and for 4 weeks on integration of proliferating cells into NeuN immunoreactive mature neurons in the dentate gyrus. Note that DCX immunoreactive neuroblasts in the Cold1W and Cold4W groups are numerous and have complex dendrites when compared with that in the CON Cold1W and CON Cold4W groups, respectively **(A,E)**. However, in the CL1W and CL4W groups, the distribution pattern and the number of DCX immunoreactive neuroblasts are similar to that in the CON CL1W and CON CL4W groups **(A,E)**. BrdU and DCX double positive cells are abundant in the Cold1W group when compared with that in the CON Cold1W group **(C)**. Similarly, BrdU and NeuN double positive cells are abundantly observed in the Cold4W group when compared with that in the CON Cold4W group **(D)**. However, there are no significant differences on the distribution pattern and number of BrdU and DCX double positive cells in the dentate gyrus between the CON CL1W and CL1W groups or the BrdU and NeuN double positive cells in the CON CL4W and CL4W groups **(C,G)**. GCL, granule cell layer; MoL, molecular layer; PoL, polymorphic layer. Quantitative analysis of DCX immunoreactive neuroblasts on the differentiation of proliferating cells into DCX immunoreactive neuroblasts (after 1 week cold exposure or CL 316,243 treatment) and on integration of proliferating cells into NeuN immunoreactive mature neurons (after 4 weeks cold exposure or CL 316,243 treatment) per section in the CON Cold1W, Cold1W, CON Cold4W, Cold4W, CON CL1W, CL1W, CON CL4W, and CL4W groups **(B,D,F,H)** (*n* = 5 per group); ^∗^ indicates a significant difference when compared with the CON group (*p* < 0.05); and ^∗∗^ indicates a significant difference when compared with the CON group (*p* < 0.01). Data are shown as mean + SEM. Black scale bar = 100 μm and green scale bar = 25 μm.

To observe differentiated neuroblasts among NSCs, DCX immunohistochemical staining was conducted. In all groups, the cytoplasms of DCX immunoreactive neuroblasts w observed in the subgranular zone of the dentate gyrus, and their dendrites extended into the molecular layer of the dentate gyrus. The Cold1W group had a higher number of DCX immunoreactive neuroblasts and more complex DCX immunoreactive fibers when compared with the CON Cold1W group (Figure 4A). The mean number of DCX immunoreactive neuroblasts was 162.00 ± 7.07 and 216.40 ± 5.59 in the CON Cold1W and Cold1W mice, respectively (Figure 4B). However, there were no changes in DCX immunoreactive cell population between the CON CL1W and CL1W groups (Figure 4A). The mean number of DCX immunoreactive neuroblasts were 154.00 ± 5.71 in the CON CL1W mice and 148.20 ± 2.41 in the CL1W mice (Figure 4B). In the number of DCX positive cells after cold challenge or CL 316,243 treatment for 1 week, statistical data of between groups were *F*(3.20) = 24.836 and *p* < 0.01. In addition, the Cold4W group showed a higher population of DCX immunoreactive neuroblasts in the dentate gyrus when compared with the CON Cold4W group (Figure 4E). The mean number of DCX immunoreactive neuroblasts was 129.40 ± 3.75 and 174.00 ± 4.76 in the CON Cold4W and Cold4W mice, respectively (Figure 4F). In addition, the number and distribution pattern of DCX immunoreactive neuroblasts were similar in the CON CL4W and CL4W groups (Figure 4E). The mean number of DCX immunoreactive neuroblasts was 121.00 ± 4.71 in the CON CL 4W mice and 126.40 ± 3.61 in the CL 4W mice (Figure 4F). In the number of DCX positive cells after cold challenge or CL 316,243 treatment for 4 weeks, statistical data of between groups were *F*(3.20) = 33.197 and *p* < 0.01.

### Cold Challenge, Not CL 316,243 Treatment, for 1 Week Increased Differentiation of Newborn Cells Into Neuroblasts in the Hippocampus

To elucidate the differentiation of newly generated cells into neuroblasts and mature neurons, BrdU and DCX double immunofluorescent staining was conducted in the dentate gyrus 1 week after cold challenge or CL 316,243 treatment. In all groups, BrdU and DCX double positive cells were observed in the subgranular zone and granule cell layer of the dentate gyrus (Figure 4C). There were different population levels of BrdU and DCX double positive cells in the Cold1W and CON Cold1W groups (Figure 4C). The mean number of BrdU and DCX double positive cells were 26.20 ± 1.52 and 46.40 ± 2.91 in the CON Cold1W and Cold1W mice, respectively (Figure 4D). However, no remarkable changes were observed in the populations of BrdU and DCX double positive cells between the CON CL1W and CL1W groups (Figure 4C). The mean numbers of BrdU and DCX double positive cells were 26.50 ± 2.64 in the CON CL1W mice and 26.00 ± 2.70 in the CL1W mice (Figure 4D). In the number of BrdU and DCX double positive cells, statistical data of between groups were *F*(3.20) = 9,916 and *p* < 0.01.

### Cold Challenge, Not CL 316,243 Treatment, for 4 Weeks Increased Integration of Newborn Cells Into Mature Neurons in the Hippocampus

To elucidate the integration of newly generated cells into mature neurons, BrdU and NeuN double immunofluorescent staining was conducted in the dentate gyrus at 4 weeks after cold challenge or CL 316,243 treatment. BrdU and NeuN double positive cells were mainly observed in the granule cell layer of the dentate gyrus (Figure 4G). Cold challenge for 4 weeks significantly increased the number of BrdU and NeuN double positive cells in the dentate gyrus when compared with that of the CON Cold4W group. The mean number of BrdU and NeuN double positive cells were 12.20 ± 0.66 and 21.00 ± 2.19 in the CON Cold4W and Cold4W mice, respectively (Figure 4H). However, there were no differences in populations of BrdU and NeuN double positive cells between the CON CL4W and CL4W groups (Figure 4G). The mean number of BrdU and NeuN double positive cells were 12.60 ± 1.29 in the CON CL4W mice and 12.60 ± 0.93 in the CL 4W mice (Figure 4H). In the number of BrdU and NeuN double positive cells, statistical data of between groups were *F*(3.20) = 9,402 and *p* < 0.01.

### Cold Challenge, Not CL 316,243 Treatment, for 1 Week or 4 Weeks Changes AHN-Related Gene, Signal Transduction Gene, and β-Adrenergic Receptor Gene Expression in the Hippocampus

**FIGURE 5:**
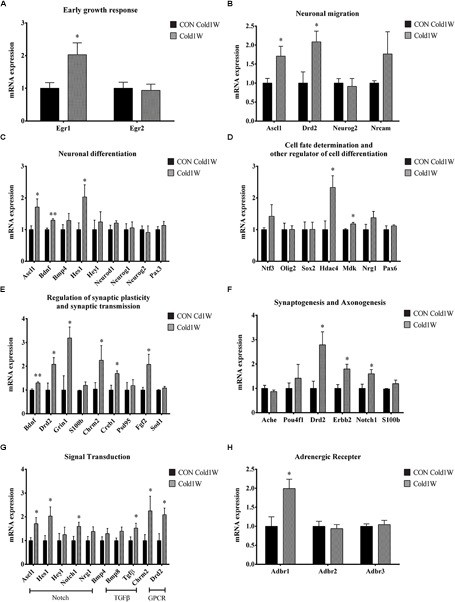
Effects of 1 week cold challenge on the expression levels of mRNA related to AHN, AHN-related signal transduction, and β-adrenergic receptor. Cold challenge for 1 week increased several genes related to **(A)** early growth response, **(B)** neuronal migration, **(C)** neuronal differentiation, **(D)** cell fate determination and other regulator of cell differentiation, **(E)** regulation of synaptic plasticity and synaptic transmission, **(F)** synaptogenesis and axonogenesis, **(G)** AHN-related to signaling transduction and **(H)** β-adrenergic receptor. ^∗^ indicates a significant difference when compared with the CON group (*p* < 0.05); ^∗∗^ indicates a significant difference when compared with the CON group (*p* < 0.01). Data are shown as mean + SEM.

**FIGURE 6:**
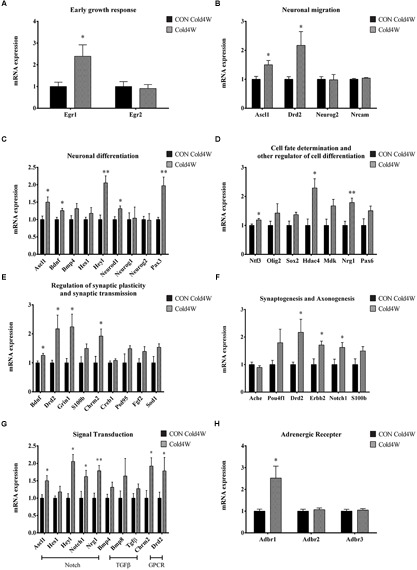
Effects of 4 weeks cold challenge on the expression levels of mRNA related to AHN, AHN-related signal transduction, and β-adrenergic receptor. Cold challenge for 4 weeks increases several genes related to **(A)** early growth response, **(B)** neuronal migration, **(C)** neuronal differentiation, **(D)** cell fate determination and other regulator of cell differentiation, **(E)** regulation of synaptic plasticity and synaptic transmission, **(F)** synaptogenesis and axonogenesis, **(G)** AHN-related to signaling transduction and **(H)** β-adrenergic receptor. ^∗^ indicates a significant difference when compared to the CON group (*p* < 0.05); and ^∗∗^ indicates a significant difference when compared with the CON group (*p* < 0.01). Data are shown as mean + SEM.

To identify the effects of cold challenge and CL 316,243 treatment for 1 week or 4 weeks on AHN-related gene, signal transduction gene, and β-adrenergic receptor gene expression, real-time PCR was conducted for these candidate genes. The control gene, 36B4, and ΔΔCt value did not show any significant changes in the hippocampal homogenates after cold challenge or CL 316,243 treatment. CL 316,243 treatment did not show any significant changes in AHN-related gene, signal transduction gene, and β-adrenergic receptor gene expression (Supplementary Figures S1, S2). However, cold challenge for 1 week or 4 weeks increased AHN-related gene expression levels in the hippocampal homogenates when compared with that in the control group. Cold challenge for 1 and 4 weeks elevated the following genes: early growth response 1 (Egr1) for immediate early response (Figure 5A, 6A); Achaete-scute homolog 1 (Ascl1) and dopamine receptor D2 (Drd2) for neuronal migration (Figure 5B, 6B); Ascl1 and BDNF for neuronal differentiation (Figure 5C, 6C); histone deacetylase 4 (Hdac4) for cell fate determination and other regulators of cell differentiation (Figure 5D); adenosine A1 receptor (Adora1), apolipoprotein E (Apoe), BDNF, and Drd2 for regulation of synaptic plasticity and synaptic transmission (Figure 5E, 6E); and Drd2, Erb-B2 receptor tyrosine kinase 2 (Erbb2), and Notch1 for synaptogenesis and axonogenesis (Figure 5F, 6F).

In contrast, cold challenge for 1 week, not 4 weeks, elevated the following genes: Hes family bHLH transcription factor 1 (Hes1) for neuronal differentiation (Figure 5C); Midkine (Mdk) for cell fate determination and other regulators of cell differentiation (Figure 5D); cAMP responsive element binding protein 1 (Creb1) and fibroblast growth factor 2 (Fgf2) for regulation of synaptic plasticity and synaptic transmission (Figure 5E).

Cold challenge for 4 weeks, not 1 week, elevated the following genes: Hairy/enhancer-of-split related with YRPW motif-like protein (Heyl), neurogenic differentiation 1 (Neurod1), and paired box gene 3 (Pax3) for neuronal differentiation (Figure 6C); neurotrophin 3 (Ntf3) and Neuregulin 1 (Nrg1) for cell fate determination and other regulators of cell differentiation (Figure 6D); Glutamate ionotropic receptor *N*-methyl-D-aspartate type subunit 1 (Grin1) and cholinergic receptor muscarinic 2 (Chrm2) for regulation of synaptic plasticity and synaptic transmission (Figure 6E).

**FIGURE 7:**
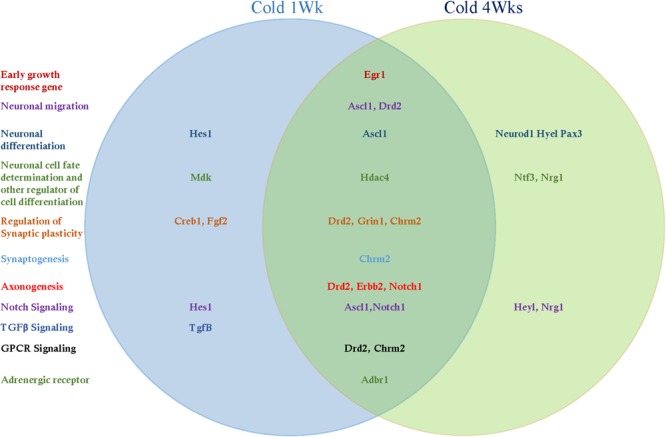
The list of hippocampal mRNAs that significantly changed after cold challenge for 1 week or 4 weeks.

Adult hippocampal neurogenesis-related signal transduction genes were categorized into three subgroups: notch, transforming growth factor-β (Tgf-β), and G protein-coupled receptor (GPCR) signaling. Cold challenge for 1 week or 4 weeks increased gene expression of the following: Ascl1 and Notch1 expression for notch signaling; and Chrm2 and Drd2 for GPCR signaling (Figure 5G, 6G). Cold challenge for 1 week, not 4 weeks, increased gene expressions of Hes1 for notch signaling and Tgf-β for Tgf-β signaling (Figure 5G). In contrast, cold challenge for 4 weeks, not 1 week, elevated Hey1 and Nrg1 expressions for Notch signaling (Figure 6G). In β-adrenergic receptor expression, adrenergic beta receptor 1 (Adbr1) was increased after 1 week or 4 weeks cold challenge (Figure 5H, 6H). A list of mRNAs changed is summarized in Figure 7 and *t*- and *p*-values after cold challenge or CL 316,243 treatment are shown in Supplementary Table S1.

## Discussion

Many studies have found that cold challenge or CL 316,243 treatment can cause beigeing effects, metabolic improvements, and therapeutic potentials for metabolic disorders (Mirbolooki et al., 2011; Labbe et al., 2016). To observe the effects of cold challenge or CL 316,243 treatment on physiological parameters, body weight and body weight gain were assessed. In the control groups, body weight steadily increased with age during the experimental period. However, cold challenge or CL 316,243 treatment for 1 week or 4 weeks significantly decreased body weight gain when compared with those in the respective control groups. In addition, cold challenge or CL 316,243 treatment caused beigeing effects in iWAT although the effects seen with CL 316,243 administration were more potent. The same effect pattern was observed in eWAT, based on hematoxylin and eosin staining. Beigeing effects were quantitatively confirmed by UCP-1 immunofluorescent staining. UCP-1 plays a key role in thermogenesis through BAT-mediated adaptive non-shivering (Kopecky et al., 1995) and the activation of UCP-1 in WAT can promote beigeing to prevent diet-induced obesity (Schulz and Tseng, 2013; Dempersmier et al., 2015). UCP-1 immunofluorescence was significantly increased in BAT and iWAT after cold challenge or CL 316,243 treatment for 1 week or 4 weeks when compared with those in the respective control groups. Similarly, UCP-1 immunoreactivity was significantly increased in the CL 316,243 treated groups when compared with that in the cold challenge group. These results suggest that both cold challenge and CL 316,243 treatment decreases body weight gain and induces beigeing effects in iWAT. This result is consistent with a previous study that showed that cold challenge or CL 316,243 administration, via similar regulating pathways, induced the activation of BAT and the formation of BeAT (Yao et al., 2017).

Cold challenge causes metabolic improvement by activating thermogenesis in adipose tissue (Harms and Seale, 2013; Jeremic et al., 2017). Many studies have demonstrated that cold challenge improves metabolic phenotypes with beigeing effects and the present study confirmed that cold challenge increased beigeing effects in iWAT. However, in the hippocampus, cold challenge is poorly understood, and controversial results have been reported. Cold challenge showed improvement in spatial learning scores and behavioral tests, but paradoxically with LTP impairment (Elmarzouki et al., 2014). The purpose of this study was to investigate the relationship between cold challenge and changes in AHN and its related genes. In the hippocampus, cold challenge increased NPCs, proliferating cells, differentiated neuroblasts, and surviving mature neurons with increased mRNA expression related to AHN. Contrary to enhanced AHN after cold challenge, CL 316,243 treatment for 1 week or 4 weeks had no effect on NPCs, proliferating cells, differentiated neuroblasts, lineage of NSCs, and surviving mature neurons. However, direct injection of a β3-adrenergic receptor agonist BRL37344 into the hippocampus increased proliferating NSCs in the subgranular zone via norepinephrine (NE) signaling (Jhaveri et al., 2010). In addition, intraperitoneal injection of isoproterenol, another type of β-adrenergic receptor agonist, elevated NPCs, while inhibition of β-adrenergic receptor by propranolol decreased NPCs and neuroblasts (Jhaveri et al., 2014). This caused NSCs and lineage of NSCs to be influenced by β-adrenergic receptor agonists and their signaling. However, the β3 specific adrenergic agonist, CL 316,243, administration via subcutaneous injection had no effect on proliferating cells and lineage of NSCs. This discrepancy may be closely related to permeability to the blood–brain barrier. These results suggest that BAT and beigeing of adipose tissue induced by CL 316,243 administration have no effects on AHN and its related gene expression. Outside the brain, CL 316,243 is known to have anti-diabetic effects in genetic rodent models of diabetes (Liu et al., 1998; Ghorbani et al., 2012). It also causes adipokine normalization in KKAy mice and obese Zucker diabetic fatty rats (Ghorbani and Himms-Hagen, 1998; Fu et al., 2008). Collectively, these results suggest that beigeing in normal animals have no effect on AHN and its related gene expression, although the observed weight gain was significantly decreased.

In the present study, cold challenge for 1 week or 4 weeks increased many AHN-related genes in the hippocampus. Egr1, also known as Zif/268, was increased after cold challenge. Egr1 has a role in the maturation and survival of newborn neurons, along with hippocampal functional maturation, such as LTP and long-term memory formation (Richardson et al., 1992; Veyrac et al., 2013). Cold challenge also increased AHN-related genes, such as Ascl1 and Drd2, which are closely related to neuronal migration, differentiation, and cell fate determination. Ascl1 is a pro-neural transcription factor that activates proliferation and differentiation of NPCs and promotes local chromatin accessibility during neurogenesis (Raposo et al., 2015). In addition, the Ascl1-expressed lineage of NSCs has long-term neurogenic potential in the subgranular zone of the adult mouse brain (Kim et al., 2011). In the present study, Drd2 expression was increased after cold challenge. Drd1 and Drd2 are expressed in mouse hippocampal neurons (Gangarossa et al., 2012); the dopaminergic pathways in hippocampal function are well known in the novel information process and synaptic plasticity (Lisman and Grace, 2005; Morice et al., 2007). Drd2 is expressed in GABAergic interneurons in the dorsal hippocampus (Puighermanal et al., 2015), which has regulatory functions in AHN (Ge et al., 2007; Pallotto and Deprez, 2014). In addition, Drd2 could control modulation of mossy cells in the dentate gyrus (Etter and Krezel, 2014). Cold challenge increased Drd2 mRNA expression in the hippocampus, and this may be a possible mechanism of cold challenge that promotes AHN via the activation of Drd2 and the GABAergic system in the dentate gyrus.

HDAC4 is a member of class IIa in the HDAC family (Bertos et al., 2001); HDAC4 lacking mouse brains show abnormalities in the hippocampus, and as it regulates neuronal cell fate determination (Majdzadeh et al., 2008). HDAC4 is one of the major components of synaptic plasticity in the hippocampus where it functions in neuronal chromatin remodeling and regulation of transcription factors (Sando et al., 2012). In addition, HDAC4 is a positive regulator of hippocampus-dependent learning and memory and behavioral results (Kim et al., 2012). In the present study, cold challenge increased mRNA expression of HDAC4, indicating that cold challenge caused HDAC4-related enhancement of AHN and hippocampal function.

Genes related to synaptic plasticity, Drd2, Grin1, and Chrm2, were increased after cold challenge. The dopaminergic pathway regulates the information process and synaptic plasticity in the hippocampus (Lisman and Grace, 2005; Morice et al., 2007). Grin1 transgenic mice showed that Grin1 is an essential factor in the acquisition of spatial memories (Tsien et al., 1996). Cholinergic projections play key roles in the proliferation and differentiation of NSCs at neurogenic niches in the hippocampus (Asrican et al., 2016). The Chrm2 gene is thought to play a role in cognitive processes (Comings et al., 2003). Muscarinic acetylcholine receptors activate signaling pathways for modulating synaptic plasticity from regulation (Levey et al., 1991; Volpicelli and Levey, 2004). Cold challenge exhibited increases in the expression of Drd2, Grin1, and Chrm2, indicating that cold challenge has effects on synaptic plasticity.

An important finding in our study was the increased levels of gene expression of the notch, GPCR, and Adbr1 pathways in the hippocampus of the Cold 1W and Cold 4W groups. In signal transduction, cold challenge increased genes categorized in notch and GPCR signaling. Notch1, as a postsynaptic receptor, has functional interactions with *N*-methyl-D-aspartate receptors, and it is an important regulator of synaptic plasticity and memory formation (Brai et al., 2015). In addition, Ascl1 has a strong correlation with the notch signaling pathway (Raposo et al., 2015). This increased levels of Notch1 and Ascl1 in notch signaling showed that cold challenge has a relationship with the notch signaling pathway. GPCR signaling underlies the basic physiology in signal transduction and the majority of mammalian GPCRs are related to central nervous system (CNS) activity (Brugarolas et al., 2014). The mechanisms of GPCR signaling are essential to the processes of neurotransmission, cellular growth, proliferation, and differentiation (Luttrell, 2008). In AHN, GPCR signaling is thought to support the adult neurogenesis process and is considered a therapeutic target for adult NSCs (Doze and Perez, 2012). The Adbr1, Chrm2, and Drd2 genes belong to the family of G protein-coupled family A receptors (Jassal et al., 2010). The increases in Drd2 and Chrm2 show that cold challenge triggers Drd2 and Chrm2 gene activation in the GPCR family A receptor, and the GPCR activation might contribute to the enhanced AHN process.

Norepinephrine and the adrenergic signaling pathway play an extremely important role in the regulation of hippocampal function. The NE receptors consist of two subtypes; α- and β-adrenergic receptors (Ahlquist, 1948). The β-adrenergic receptor has very specific effects on synaptic plasticity when compared with that of the α-adrenergic receptor (Segal et al., 1991; Kemp and Manahan-Vaughan, 2008). In addition, in the dentate gyrus, the synaptic plasticity is highly sensitive to β-adrenergic control when compared with other hippocampal subregions (Hagena et al., 2016). Memory consolidation and synaptic plasticity in the hippocampus are controlled by noradrenergic signaling (Hagena et al., 2016). Noradrenergic signaling in the hippocampus activates the β1- and β2-adrenergic receptors and facilitates phosphorylation of CREB (Hagena et al., 2016), which increases LTP by β-adrenergic receptor modulation and memory-promoting effects (O’Dell et al., 2015). Activation of β1 adrenergic receptor signaling enhances hippocampal network activity in the CA3 and shows correlation with NE signaling with cognitive process (Jurgens et al., 2005). In the present study, cold challenge for 1 or 4 weeks elevated the expression of Adbr1 mRNA, indicating that this activates β-adrenergic signaling in the hippocampus. Increased Adbr1 expression in the present study can be one of the possible mechanisms to enhance AHN after cold challenge.

In summary, ambient cold challenge decreases body weight gains and has beigeing effects. In addition, cold challenge increases phenotypes of AHN and its related mRNA gene expression. In contrast, beigeing induced by CL 316,243 treatment did not show any significant increases in AHN and its related genes. These results suggest that inducing peripheral beigeing may not be sufficient to induce AHN and that cold challenge can promote AHN through increasing AHN-related gene expression, not through beigeing effects in BAT and iWAT.

## Author Contributions

JK, KH, WK, HJ, SN, DY, IH, JS, and YY conceived the study. JK, SN, DY, and IH designed the study. JK wrote the manuscript. JS and YY edited the manuscript. JK, KH, WK, and HJ conducted the animal experiments. JK and KH measured real-time PCR. IH, JS, and YY participated in designing and discussing the study. All authors read and approved the final manuscript.

## Conflict of Interest Statement

The authors declare that the research was conducted in the absence of any commercial or financial relationships that could be construed as a potential conflict of interest.

## Supplementary Material

FIGURE S1 Effects of 1 week CL 316,243 treatment on the expression levels of mRNA related to AHN, AHN-related signal transduction, and β-adrenergic receptor. There were no significant differences in gene expression levels of **(A)** early growth response, **(B)** neuronal migration, **(C)** neuronal differentiation, **(D)** cell fate determination and other regulators of cell differentiation, **(E)** regulation of synaptic plasticity and synaptic transmission, **(F)** synaptogenesis and axonogenesis, **(G)** AHN-related to signaling transduction, and **(H)** β-adrenergic receptor.

FIGURE S2 Effects of 4 weeks CL 316,243 treatment on the expression levels of mRNA related to AHN, AHN-related signal transduction, and β-adrenergic receptor. There were no remarkable changes in mRNA expression levels of **(A)** early growth response, **(B)** neuronal migration, **(C)** neuronal differentiation, **(D)** cell fate determination and other regulator of cell differentiation, **(E)** regulation of synaptic plasticity and synaptic transmission, **(F)** synaptogenesis and axonogenesis, **(G)** AHN-related to signaling transduction, and **(H)** β-adrenergic receptor.

TABLE S1 *t*- and *p*-values from statistical analysis of qRT-PCR data.
