# Supplementary material for: Adult Hippocampal Neurogenesis Can Be Enhanced by Cold Challenge Independently From Beigeing Effects
Source: Front Neurosci. 2019 Mar 5;13:92. doi: 10.3389/fnins.2019.00092 (PMC6411820; doi:10.3389/fnins.2019.00092)
Supplement: TABLE S1 — t- and p-values from statistical analysis of qRT-PCR data. [file Table_1.docx]

|  | gene | *t* value | *p* value | gene | *t* value | *p* value |
| --- | --- | --- | --- | --- | --- | --- |
| Cold 1 week | Egr1 | 2.577 | 0.033 | Egr2 | 0.757 | 0.471 |
|  | Ascl | 2.490 | 0.038 | Drd2 | 2.683 | 0.028 |
|  | Neurog2 | 0.832 | 0.429 | Nrcam | 1.300 | .0262 |
|  | Bdnf | 4.387 | 0.002 | Bmp4 | 1.071 | 0.316 |
|  | Hes1 | 2.343 | 0.047 | Heyl | 0.563 | 0.589 |
|  | Neurod1 | 1.321 | 0.223 | Neurog1 | 0.211 | 0.838 |
|  | Pax3 | 0.858 | 0.416 | Ntf3 | 1.139 | 0.288 |
|  | Olig2 | 0.013 | 0.990 | Sox2 | 0.031 | 0.976 |
|  | Hdac4 | 2.886 | 0.020 | Mdk | 3.223 | 0.012 |
|  | Nrg1 | 1.437 | 0.189 | Pax6 | 0.971 | 0.360 |
|  | Bdnf | 4.387 | 0.002 | Grin1 | 2.888 | 0.020 |
|  | S100b | 1.506 | 0.205 | Chrm2 | 1.792 | 0.111 |
|  | Creb1 | 2.946 | 0.019 | Psd95 | 0.457 | 0.660 |
|  | Fgf2 | 2.479 | 0.038 | Sod1 | 1.185 | 0.270 |
|  | Ache | 0.920 | 0.384 | Pou4f1 | 0.686 | 0.512 |
|  | Erbb2 | 3.257 | 0.012 | Notch1 | 2.436 | 0.041 |
|  | Bmp8 | 1.936 | 0.089 | TgfB | 2.033 | 0.076 |
|  | Adbr1 | 2.849 | 0.022 | Adbr2 | 0.368 | 0.723 |
|  | Adbr3 | 0.333 | 0.748 |  |  |  |
|  |  |  |  |  |  |  |
|  |  |  |  |  |  |  |
|  |  |  |  |  |  |  |
|  |  |  |  |  |  |  |
|  |  |  |  |  |  |  |
|  |  |  |  |  |  |  |

|  | gene | *t* value | *p* value | gene | *t* value | *p* value |
| --- | --- | --- | --- | --- | --- | --- |
| Cold 4 weeks | Egr1 | 2.473 | 0.039 | Egr2 | 0.317 | 0.760 |
|  | Ascl | 2.811 | 0.023 | Drd2 | 2.443 | 0.040 |
|  | Neurog2 | 1.165 | 0.278 | Nrcam | 1.254 | 0.245 |
|  | Bdnf | 2.789 | 0.024 | Bmp4 | 1.696 | 0.128 |
|  | Hes1 | 1.003 | 0.345 | Heyl | 4.405 | 0.002 |
|  | Neurod1 | 2.725 | 0.026 | Neurog1 | 0.105 | 0.919 |
|  | Pax3 | 3.801 | 0.005 | Ntf3 | 2.807 | 0.023 |
|  | Olig2 | 1.194 | 0.267 | Sox2 | 2.157 | 0.063 |
|  | Hdac4 | 3.319 | 0.011 | Mdk | 2.181 | 0.061 |
|  | Nrg1 | 3.775 | 0.005 | Pax6 | 1.825 | 0.105 |
|  | Bdnf | 2.789 | 0.024 | Grin1 | 2.688 | 0.028 |
|  | S100b | 1.912 | 0.092 | Chrm2 | 2.838 | 0.022 |
|  | Creb1 | 0.986 | 0.353 | Psd95 | 1.507 | 0.170 |
|  | Fgf2 | 1.777 | 0.113 | Sod1 | 2.169 | 0.062 |
|  | Ache | 0.978 | 0.357 | Pou4f1 | 1.530 | 0.165 |
|  | Erbb2 | 2.788 | 0.024 | Notch1 | 2.338 | 0.048 |
|  | Bmp8 | 1.171 | 0.275 | TgfB | 1.426 | 0.192 |
|  | Adbr1 | 2.729 | 0.026 | Adbr2 | 0.561 | 0.590 |
|  | Adbr3 | 0.439 | 0.672 |  |  |  |
|  |  |  |  |  |  |  |
|  |  |  |  |  |  |  |
|  |  |  |  |  |  |  |
|  |  |  |  |  |  |  |
|  |  |  |  |  |  |  |
|  |  |  |  |  |  |  |

|  | gene | *t* value | *p* value | gene | *t* value | *p* value |
| --- | --- | --- | --- | --- | --- | --- |
| CL 316,243  1 week | Egr1 | 0.231 | 0.823 | Egr2 | 0.304 | 0.769 |
|  | Ascl | 0.306 | 0.768 | Drd2 | 0.053 | 0.959 |
|  | Neurog2 | 0.061 | 0.953 | Nrcam | 0.558 | 0.592 |
|  | Bdnf | 1.307 | 0.228 | Bmp4 | 1.023 | 0.336 |
|  | Hes1 | 0.032 | 0.975 | Heyl | 0.391 | 0.706 |
|  | Neurod1 | 0.542 | 0.603 | Neurog1 | 0.005 | 0.996 |
|  | Pax3 | 0.348 | 0.737 | Ntf3 | 0.582 | 0.576 |
|  | Olig2 | 0.310 | 0.764 | Sox2 | 0.063 | 0.951 |
|  | Hdac4 | 0.420 | 0.686 | Mdk | 0.862 | 0.414 |
|  | Nrg1 | 0.151 | 0.884 | Pax6 | 0.051 | 0.961 |
|  | Bdnf | 1.307 | 0.228 | Grin1 | 0.070 | 0.946 |
|  | S100b | 0.374 | 0.718 | Chrm2 | 0.553 | 0.595 |
|  | Creb1 | 0.502 | 0.629 | Psd95 | 0.312 | 0.763 |
|  | Fgf2 | 0.014 | 0.989 | Sod1 | 0.571 | 0.583 |
|  | Ache | 0.100 | 0.923 | Pou4f1 | 0.089 | 0.932 |
|  | Erbb2 | 0.492 | 0.636 | Notch1 | 0.255 | 0.805 |
|  | Bmp8 | 0.169 | 0.870 | TgfB | 0.482 | 03643 |
|  | Adbr1 | 0.197 | 0.849 | Adbr2 | 0.176 | 0.865 |
|  | Adbr3 | 0.045 | 0.965 |  |  |  |
|  |  |  |  |  |  |  |
|  |  |  |  |  |  |  |
|  |  |  |  |  |  |  |
|  |  |  |  |  |  |  |
|  |  |  |  |  |  |  |
|  |  |  |  |  |  |  |

|  | gene | *t* value | *p* value | gene | *t* value | *p* value |
| --- | --- | --- | --- | --- | --- | --- |
| CL 316,243  4 weeks | Egr1 | 0.317 | 0.759 | Egr2 | 0.741 | 0.480 |
|  | Ascl | 0.321 | 0.756 | Drd2 | 0.112 | 0.914 |
|  | Neurog2 | 0.672 | 0.521 | Nrcam | 0.666 | 0.524 |
|  | Bdnf | 1.458 | 0.183 | Bmp4 | 0.446 | 0.667 |
|  | Hes1 | 0.059 | 0.954 | Heyl | 0.387 | 0.709 |
|  | Neurod1 | 0.777 | 0.460 | Neurog1 | 0.679 | 0.517 |
|  | Pax3 | 0.586 | 0.574 | Ntf3 | 0.548 | 0.598 |
|  | Olig2 | 0.558 | 0.592 | Sox2 | 0.102 | 0.921 |
|  | Hdac4 | 0.792 | 0.451 | Mdk | 1.811 | 0.108 |
|  | Nrg1 | 0.531 | 0.610 | Pax6 | 1.601 | 0.148 |
|  | Bdnf | 1.458 | 0.183 | Grin1 | 0.202 | 0.845 |
|  | S100b | 2.269 | 0.053 | Chrm2 | 0.252 | 0.807 |
|  | Creb1 | 1.072 | 0.315 | Psd95 | 0.275 | 0.790 |
|  | Fgf2 | 0.968 | 0.361 | Sod1 | 1.942 | 0.088 |
|  | Ache | 0.805 | 0.444 | Pou4f1 | 0.370 | 0.721 |
|  | Erbb2 | 0.622 | 0.552 | Notch1 | 0.561 | 0.590 |
|  | Bmp8 | 0.452 | 0.663 | TgfB | 0.791 | 0.451 |
|  | Adbr1 | 0.258 | 0.803 | Adbr2 | 0.288 | 0.781 |
|  | Adbr3 | 0.250 | 0.843 |  |  |  |
|  |  |  |  |  |  |  |
|  |  |  |  |  |  |  |
|  |  |  |  |  |  |  |
|  |  |  |  |  |  |  |
|  |  |  |  |  |  |  |
|  |  |  |  |  |  |  |
